# Combinatorial Method/High Throughput Strategies for Hydrogel Optimization in Tissue Engineering Applications

**DOI:** 10.3390/gels2020018

**Published:** 2016-06-08

**Authors:** Laura A. Smith Callahan

**Affiliations:** 1 Vivian L. Smith Department of Neurosurgery & Center for Stem Cells and Regenerative Medicine McGovern Medical School at the University of Texas Health Science Center at Houston, Houston, TX 77030, USA; 2 Department of Nanomedicine and Biomedical Engineering, McGovern Medical School at the University of Texas Health Science Center at Houston, Houston, TX 77030, USA; laura.a.smithcallahan@uth.tmc.edu; Tel.: +01-713-500-3431

**Keywords:** tissue engineering, hydrogel, high throughput, design of experiment, array and continuous gradient

## Abstract

Combinatorial method/high throughput strategies, which have long been used in the pharmaceutical industry, have recently been applied to hydrogel optimization for tissue engineering applications. Although many combinatorial methods have been developed, few are suitable for use in tissue engineering hydrogel optimization. Currently, only three approaches (design of experiment, arrays and continuous gradients) have been utilized. This review highlights recent work with each approach. The benefits and disadvantages of design of experiment, array and continuous gradient approaches depending on study objectives and the general advantages of using combinatorial methods for hydrogel optimization over traditional optimization strategies will be discussed. Fabrication considerations for combinatorial method/high throughput samples will additionally be addressed to provide an assessment of the current state of the field, and potential future contributions to expedited material optimization and design.

## 1. Introduction

The extracellular matrix (ECM) was once thought to be inert [1], but has been found to significantly influence cellular behavior [2,3]. The native ECM of most tissues in the body is a highly hydrated, viscoelastic network of proteoglycans, glycoaminoglycans, and proteins, which provide mechanical, chemical and physical cues to guide cell behavior and tissue homeostasis [1]. A similar progression from being viewed as inert to possessing the potential to guide cellular behavior through compositional blending, tailoring material properties and inclusion of bioactive signaling has occurred with biomaterials [4,5,6,7,8]. Our growing understanding of ECM function in directing cellular behavior has driven this change and spurred a movement to emulate key aspects of the ECM with biomaterials. The complexity of our emulation strategies have advanced with both our biological understanding and technical capabilities. Determining the minimal number of factors needed to achieve the desired cellular behavior outcome remains the major design objective for matrices. Due to their high aqueous content and tailorable material properties that can mimic native ECM properties in many tissues, hydrogels have become widely used as the base matrix in these ECM emulation strategies for tissue engineering platforms [9].

The versatility of hydrogels leads to a number of parameters that can be altered to meet the design criteria for a given tissue engineering application. For instance, hydrogels can be composed of synthetic polymers, natural polymers, or a hybrid of the two [10,11,12]. The gelation mechanism can be varied from photopolymerization to click chemistry, to Michael addition, to physical entanglement, *etc.* [10,11,13,14]. Bioactive signaling molecules can be tethered or released from the hydrogel at various concentrations and rates [12,15,16,17]. Even the topography, porosity and crosslink density can be manipulated [18,19,20]. The sheer number of design options for hydrogel construction has traditionally led to an ad hoc trial and error approach to their testing and optimization for clinical use. This has led to a limited understanding of key hydrogel design parameters that affect cellular behavior, slowed the development process and reduced the number of hydrogel based treatments reaching clinical application.

Combinatorial method/high throughput strategies, which have long been used in pharmaceutical development to screen multiple molecules in parallel [21], offer the potential to expedite hydrogel development for clinical tissue engineering applications and our understanding of cell-biomaterial interfaces. These strategies allow for efficient exploration of a large compositional space [22]. They are particularly useful as the ability to theoretically predict cellular response to hydrogels is currently limited, meaning brute force experimentation is necessary to develop enough base information for development of robust predictive models [22]. If coupled with sample miniaturization and automation, these strategies can reduce the overall cost of the research in terms of reagents and manpower. The number of cells needed to obtain the information can also be reduced, which is particularly helpful with rare or difficult to culture cell types, compared to traditional approaches.

Although many combinatorial method/high throughput strategies have been developed [23,24,25], few are suitable for hydrogel optimization in tissue engineering applications as both two- and three-dimensional cell culture are necessary and multiple types of design parameters ranging from composition to functionalization with bioactive signaling molecules must be examined. Currently, the combinatorial/high throughput strategies that have been applied to hydrogel optimization are design of experiment (DOE), arrays and continuous gradients. In this review, the basics of each of the applied methods will be covered along with the advantages and drawbacks. Since these strategies could ultimately be used in combination for hydrogel optimization, general sample design considerations will be covered as well.

## 2. Design of Experiment

DOE is a statistical software approach used to identify the best parameters for a desired outcome. In this method, each input is entered into a predictive model in order to determine the combination of factors necessary to achieve the desired outcome. Normally multiple rounds of experiments are necessary to optimize the material and achieve the cellular response desired as the data from the current round of optimizations is added to the predictive model to refine the predictions for the next round of testing. The number of rounds is dependent on the number of inputs, outcomes and the design approach, either full or fractional factorial [26]. Full factorial design covers all the possible combinations of the inputs. This requires lots of resources (manpower and reagents), which makes it impractical for many studies. Fractional design runs the minimum number of experiments to identify the desired outcome, so only a portion of the possible combinations are run. In this case, the needed resources are reduced, but the potential to misidentify or completely miss effects are possible. Even with reduced resource requirements, the number of samples necessary for the optimization can still be large in order to identify the main effects and crosstalk interactions. One study optimizing 35 test conditions, ranging from cell density and ratio of different cell types to hydrogel composition and thickness, for human umbilical vein endothelial cell (HUVEC) culture required 200 samples to identify a paracrine effect between vascular network formation and osteogenically differentiated human mesenchymal stem cells (hMSC) and create a three dimensional hydrogel model for further biological study of this effect [27].

Fractional DOE design has been used to optimize Arg-Gly-Asp (RGD), Tyr-Ile-Gly-Ser-Arg (YIGSR) and Ile-Lys-Val-Ala-Val (IKVAV) peptide concentration simultaneously in hydrogels instead of more traditional approach of optimizing one peptide at a time. One study optimizing the RGD (8 mM), YIGSR (0 mM) and IKVAV (3 mM) peptide concentrations for HUVEC culture in nanofibrous self-assembly peptide scaffolds identified an antagonistic relation between RGD and YIGSR, contrary to previous reports that had not utilized a high throughput strategy [28]. Another study optimized the RGD (100 μM), YIGSR (48 μM) and IKVAV (300 μM) peptide concentrations to promote human induced pluripotent stem cells survival during neural differentiation, increasing the number of neural progenitors available for further study [29]. Both studies found simultaneous optimization yielded different optimal concentrations for each peptide with improved biological response compared to individual optimization followed by combination of those peptide concentrations into a single sample. Both studies demonstrate the power of the DOE approach to efficiently optimize multiple design parameters. In addition, its potential to reveal synergistic or detrimental effects due to parameter interactions that may not be uncovered with traditional optimization strategies. However, this efficiency comes from access to the software, which is expensive, and good predictive models. Typically, good predictive models are made using data from experiments examining the cellular response to each test parameter individually. For many design parameters and cell types, the information needed to populate the predictive model is not known and must be obtained, adding to the number of necessary experiments. As the base of knowledge regarding cellular response grows the quality of the initial predictive model will become less of an issue.

## 3. Arrays

Arrays use a number of discrete, often miniaturized, samples to optimize material properties for a desired cellular response. The strategy is compatible with a number of formats. Hydrogel arrays have been printed with soft lithography [30], direct contact printing [22] and inkjet printing [31]; injection molded in microfluidic channels [32]; held in free floating molds [33,34] and attached to glass cover slides [35]. This flexibility allows for their automated fabrication with liquid handling systems. A comparison of cell viability between automated and hand pipetted array systems has found higher cellular viability when automated fabrication was used with multiple cell types [33]. Use of automated liquid handing systems allows for larger arrays with more test conditions than could typically be fabricated by hand. One study, which utilized an automated liquid handling system to fabricate the array, examined 400 test conditions in gelatin hydrogels to study protein effects on hMSC osteogenic differentiation via mineralization of the gelatin matrix [36]. Another study assessing the effects of five different signaling types on the maintenance of pluripotency in mouse embryonic stem cells examined over 1000 test conditions [37]. However, automated liquid handling systems are expensive, which inhibits many from using them to build arrays and significantly lowers the number of test conditions examined in many studies. For comparison, one large array study fabricated by hand pipetting examined 19 test conditions with seven cell types [38]. Reductions in the number of test conditions decreases the chance that optimal conditions will be identified and that secondary relationships or interactions between test parameters will be detected. This mitigates some of the advantages of utilizing a combinatorial/high throughput approach. However, the development of graphical bar codes on polymer and ECM components offers a way to increase tested conditions in hand pipetted arrays [39], making increases in test condition numbers in manually fabricated systems practical. Eventually, the development of less expensive technologies for array fabrication, such hydrophobically created microgels, could additionally ease the burden of hand fabrication or bring the cost of automated array fabrication within the reach of more researchers [40].

Although not ideal for optimization, arrays are well suited for initial discovery of potentially advantageous hydrogel conditions. Inkjet printing coupled with a reduction-oxidation reaction allows for the addition of multiple materials to create complex formulations [31]. Using this approach, a study focused on polymer discovery examined 2280 different formulations to identify a thermally responsive polymer which would release cells upon cooling to room temperature from 37 °C [41]. Use of automated liquid handling for contact printing followed by photopolymerization is another strategy used for this type of array fabrication [22,42]. This approach was used to identify monomers capable of promoting human embryonic stem cell (hESC) differentiation on poly(hydroxyethyl methacrylate) hydrogels from over 1700 test formulations [22].

Alterations in material properties due to changes in composition have been studied using arrays. These studies have ranged from characterizations of gelation kinetics [43] to fibronectin absorption [42]. A study of the effects of polymer chemistry on hESC attachment provided enough data to create a model capable of predicting hESC attachment to novel polymers [44]. The strategy has even been used to identify the optimal hydrogel to release Lipolexe-based transfection agents for efficient transfection of cells cultured on the hydrogel surface [45]. Moving beyond standard material characterization, the flexibility of array construction has allowed for arrays with spatial patterns [46], printed on surfaces with nanofibrous architecture [47] and multiplexed test parameters due to independent patterning methodologies [48] to be fabricated and characterized. This increased fabrication complexity allows for the greater emulation of the native ECM at a structural level and the study of multiple physical stimuli on material behavior and cellular response at the same time.

Cellular response to a number of hydrogel design parameters have been examined with the array format. These studies have focused on identifying optimal hydrogel formulation [22,42,45,49,50,51], mechanical properties [35,52], hydrogel degradation [33,37,53] and bioactive signaling molecule concentration [33,35,37,52,54] to illicit a desired cellular response. The range of desired cell behaviors observed and used as the selection criteria for the optimal hydrogel has ranged from attachment [41,42,44,51,55,56,57], cellular morphology [32] and viability [35,48,53,54,58] to migration [52] and lineage choice [30,54]. As the study of cell-biomaterial interface has advanced, so has the selection of cellular behavior utilized as the material selection criteria. This has led to more advanced studies examining the effect of protein concentration on non-adherent neurosphere proliferation, quiescence and death [54], and the ability of dendritic cells to undergo phagocytosis while adhering to hydrogels to be conducted in array format [57].

Like DOE, these studies can optimize more than one parameter at a time. Simultaneous optimization of three parameters at the same time have been reported [37]. One recent study of hMSC adhesion found that changes in Young’s modulus altered cellular spreading and focal adhesion formation in response to RGD concentration, indicating an interconnection between the two signals in the cell [35]. However, a similar study of Young’s modulus and RGD concentration found the materials mechanical properties to be the major factor affecting fibrosarcoma cellular morphology and migration [52]. The conflicting results between cell types demonstrates the need to run these systematic studies for every cell type of interest as results from one cell type cannot easily be extrapolated to predict the response of another cell type. To further highlight the complexity of parameter interaction that combinatorial methods/high throughput studies can detect, a recent study by Ranga and co-workers examined the effects of Young’s Modulus, matrix degradability, tethered and released bioactive signaling molecules, and cellular density on the maintenance of pluripotency in mouse embryonic stem cells using bioinformatic analysis tools [37]. Although their work illustrated to the predominate role of leukemia inhibitory factor in this process, it identified synergistic and detrimental effects of the other test parameters on this process, which had not previously been identified.

## 4. Gradient Samples

**Figure 1:**
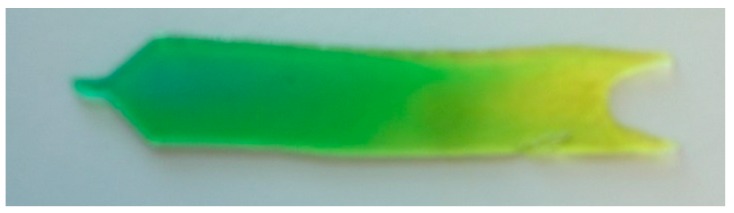
Demonstration of colormetric gradient in a polyethylene glycol hydrogel. Adapted with the permission from [73]. Copyright 2015 Elsevier.

Gradient samples can be fabricated with simple inexpensive systems comprised of pumps and molds [59]. Although elimination of the pump is possible through use of passive methods such as surface tension to drive flow through the mold [60]. Newer methods of gradient formation are less reliant on pumps for their formation as they use the mold [61], thermal cycles [61,62], or ultraviolet light exposure [63,64] to generate the gradient instead of flow. Gradient samples consist of a gradual compositional change between two or more parameters (Figure 1). However, this compositional change does not have to be linear as exponential, sigmoidal and radial gradients have been fabricated [65,66,67]. The growing inclusion of orthogonal chemistries is increasing the number of test parameter gradients, which can be overlaid in a single sample [68,69]. Due to this flexibility, variations in fabrication of gradient samples have ranged from complex microfluidics, which can directly overlay two orthogonal gradients for the study of multiple parameters at a time [70], to large gradients (6 cm by 6 cm) fabricated using a peristaltic pump drawing from two polymer reservoirs into a mold in order to create many replicates or large samples for complex analysis methods from the same gradient hydrogel [71,72].

Like arrays, gradient samples have been used to characterize changes in material properties due alterations in polymer chemistry and hydrogel formulation [74,75]. These characterization studies have been expanded to examine the linear and non-linear gradient release of growth factors and drugs from hydrogels in order to optimize release kinetics from the hydrogel for localized delivery [76,77,78,79]. Even short interfering RNA gradients have been fabricated [80], with one study demonstrating complete silencing of green fluorescent protein expression in encapsulated cells with high concentrations of incorporated short interfering RNA in the hydrogel [80].

Due to the continuously changing nature of the gradient samples, the number of distinct formulations tested cannot be directly calculated like in DOE and arrays formats. As such, isolating the optimal composition after testing can be more difficult in gradient samples than DOE and array samples. Good characterization of the gradient’s material properties is necessary for identification of the optimal test conditions to occur. It also identifies confounding factors due to material property changes in the sample other than the test parameter along the length of the gradient created due to fabrication of the gradient for the test parameter. This is important because changes in bioactive signaling inclusion and polymer composition have been shown to alter a wide range of material properties in hydrogels such as Young’s modulus, mesh size and swelling ratio [81,82,83]. Often DOE and array samples rely on formulation data to identify the optimized condition, instead of directly measuring material properties in the fabricated samples. As all of the included components may not have anchored into the hydrogel during fabrication and additional material properties may have been altered beyond the test parameter, this may not be an accurate presentation of the hydrogel environment the cells interacted with. A strategy based on direct measurement of all test and material properties in the fabricated hydrogel system will provide the most accurate determination of the optimized condition with all combinatorial method/high throughput approaches, not just gradient samples.

The complex relationship between hydrogel material properties and cellular response makes this level of characterization even more critical. Small changes in a number of factors, even some of which were unintended, may alter the observed cellular response. To illustrate this, changes in hydrogel thickness, fiber density and stiffness have been observed along polymer concentration gradients [83,84,85], each of which can alter cellular response to the hydrogel [5,86,87]. Overlapping gradients in hydrogel wettability and stiffness found an interaction between the two material properties affecting hMSC adhesion and spreading, where changes in wettability lead to alterations in the material stiffness where hMSC adhesion and spreading occurred [88]. This study demonstrates the complexity of these interactions between multiple material properties on cellular behavior. Compositional blending gradients of polymers or solvents at different ratios have been used to form porosity gradients in hydrogels [85,89,90]. Changes in porosity across the gradient have been found to alter cell cytoskeletal structure [90], which can alter later differentiation [91]. It is important to note that one study demonstrated a difference in hydrogel stiffness along with the porosity change [85], which would complicate analysis of cellular results due to the porosity change. Reaction kinetics in hydrogel photopolymerization based on ultraviolet light exposure time have been monitored [92]. Low conversion rates due to insufficient ultraviolet light exposure were associated with reduced macrophage viability and increased expression of inflammation markers by the cells [64,93]. This demonstrates that not just formulation changes, but also the efficiency of the system to completely consume the reactive elements utilized for gelation or functionalization can have significant effect on cellular response.

**Figure 2:**
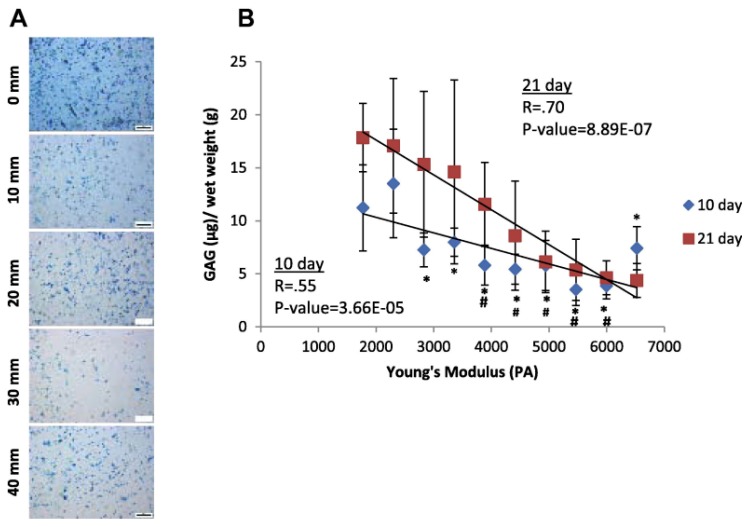
Extracellular matrix (ECM) production by human chondrocytes after 10 days of culture. Images were taken at 10 mm intervals along the length of the modulus gradient. (**A**) Whole mount Alcian blue; (**B**) sulfated gylcosaminoglycan quantification based on Alcian blue extraction shows distinct changes with position in the modulus profile at both 10 and 21 days. Scale bar 200 μm. # indicates *p* ≤ 0.05 compared with the 1700 Pa Young’s modulus gradient position; * indicates *p* ≤ 0.05 compared with the 2300 Pa Young’s modulus gradient position. R (coefficient of multiple correlation) and P-value indicate statistical results of linear regression analysis, and indicate a high confidence in the linear relationship in the data. Adapted with the permission from [83]. Copyright 2013 Elsevier.

One of the major advantages of the gradient approach over DOE and arrays in biological studies is that every possible concentration or combination of test parameters within the test range is present in the gradient hydrogel and is routinely examined. This makes the approach particularly well suited for the optimization of the test parameters. The small changes in sample composition across gradient samples have been found to affect cellular attachment [90,92,94,95,96,97], viability [27,64,72], migration [66,69,70,98,99,100] and differentiation [71,72,73,83,101,102,103,104]. To demonstrate this phenomena, Figure 2 shows a study of the effects of a Young’s modulus gradient on human chondrocyte glycosaminoglycan content. Detection of these shifts in cellular behavior are easier in gradient samples than in DOE and arrays due to reduced sample preparation, which limits sample variation effect on the biological results.

Due to the ease and flexibility of fabrication, a number of studies have utilized gradient hydrogel systems to examine the effect of material property changes spanning from studies of Young’s modulus [11,66,70,83,92,96,97,99,101,104,105,106] to bioactive signal concentration [19,22,76,77,78,79,90,94,95,100,102,107] on cellular behavior with numerous cell types. Large changes in hydrogel stiffness have been shown to affect the lineage choice of stem cells and cellular proliferation [108]. Small changes have been shown to affect cellular function, as in Figure 2, where ECM content was altered [83]. Both small and large stiffness gradients have demonstrated changes in cytoskeletal structure [62,83]. Similar changes in cytoskeletal structure have been observed in bioactive signal concentration gradients [102]. These early changes in cellular behavior due to interactions with the hydrogel could led to the differences in cell response observed at later time points [91].

Ideally, the gradients should not be sensed at the cellular level and cells respond as if in a homogenous material. However, steep gradients, which were sensed by individual cells have been observed and led to the cellular alignment along the gradient [100,107,109,110,111]. There are certain biological systems where this alignment is advantageous, for instance when used to develop a predictive model of cell migration [112], direct cellular migration down a mechanotactic gradient [61], or recapitulate a biological gradient to aid in tissue formation [113,114]. However, it is often an unintended confounding factor, which complicates the material optimization process for tissue engineering. Once identified the test range for the given parameter can easily be altered to eliminate cellular alignment along the gradient. Shifting the test parameter range within gradient samples does not require many changes to sample fabrication. The technique of altering the test parameter range has, also, been used to study regions of interest at greater resolution [73]. This allows for fine tuning of the optimized condition to maximize the desired cellular effects quickly.

Complex biological systems can be studied with a gradient approach using overlapping or sequential gradients [84,92,115]. Using overlapping gradients of nerve growth factor and neurotrophin-3 immobilized in poly(2-hydroxyethylmethacrylate) hydrogels, one study identified a synergistic, and not merely additive, effect of the proteins on chick dorsal root ganglia neurite extension [76]. A study of orthogonal Young’s Modulus and protein concentration gradients found cellular migration distance and velocity increased with increasing hydrogel Young’s modulus at low hepatocyte growth factor concentration, but that changes in Young’s modulus had no effect on migration distance and velocity at a high hepatocyte growth factor concentrations [70]. Again demonstrating the complexity of relationships among multiple biological signals, and how under the right conditions one can play a dominate role over the others in directing cellular response. Another study was able to provide real time monitoring of endothelial cells while controlling hydrogel properties, solute gradients, surface shear stress and interstitial flow through the matrix [98], helping to determine the optimal combination to spur vessel formation. Beyond high throughput analysis, these systems can be further developed to emulate tissue development and native function. These *in vitro* models can then serve as drug testing platforms or models to study human development and disease progression.

## 5. Combinatorial Method/High Throughput Sample Design Considerations

Regardless of the combinatorial method/high throughput approach used to conduct these studies, there are number of design parameters that should be considered when cell culture experiments are being conducted. The first is that there are limitations on the selection of polymers and gelation approaches, which can be utilized in these approaches. Inappropriate viscosity and gelation kinetics can lead to inconsistent sample formation. This is particularly true for three-dimensional culture systems as the cellular distribution may not be consistent throughout the sample, potentially altering cellular behavior in the sample and experimental results. Due to its robust consistent network formation and speed [66,73,93], photopolymerization has most often been used for gelation of combinatorial method/high throughput samples. However, reduction-oxidative reactions have proven suitable in certain sample fabrications for use in combinatorial method/high throughput approaches [31,41].

**Figure 3:**
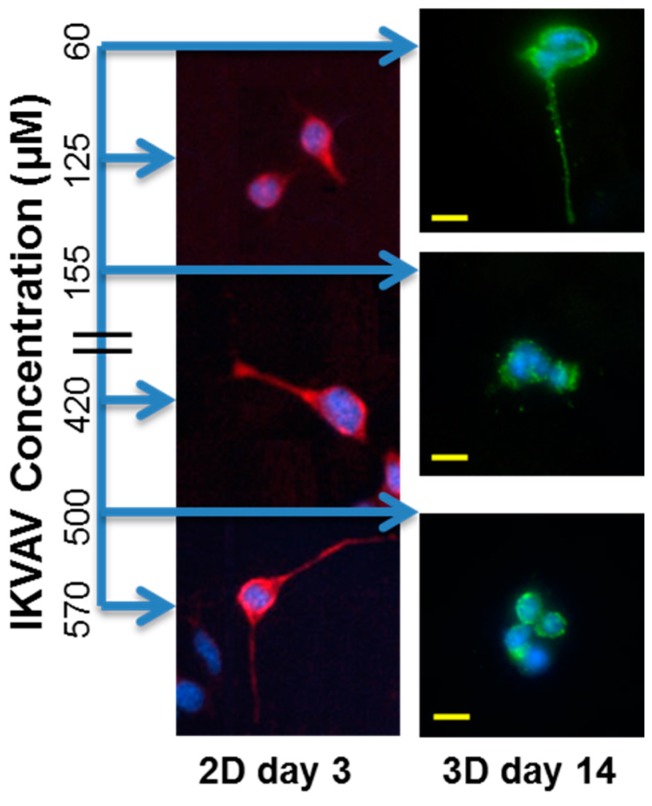
Beta III tubulin staining of neurite extension of cells in 2D (red) and 3D (green) culture with nuclear staining (blue) exposed to a continuous IKVAV gradient in polyethylene glycol hydrogels after 3 days of culture in 2D and 14 days of culture in 3D. Scale bars: 10 μm. Adapted with permission from [73]. Copyright 2015 Elsevier.

Even if cellular distribution is homogenous across the sample, major differences in the cellular environment exist between two-and three-dimensional culture that can alter the optimal hydrogel formulation [116]. This was effectively demonstrated in a recent study examining the effects of IKVAV concentration on mouse embryonic stem cell neural differentiation [73]. Not only was a significant drop in the IKVAV concentration capable of promoting neurite extension observed in three-dimensional culture compared to two-dimensional culture, but also a significant delay in neural differentiation in three-dimensional culture compared to two-dimensional culture (Figure 3). This study demonstrates that extrapolation of optimal conditions from two- to three-dimensional studies will be at best difficult. As both culture methods are useful for cellular expansion and tissue formations in tissue engineering applications, both culture methods need to continue to be studied with a systematic approach.

**Figure 4:**
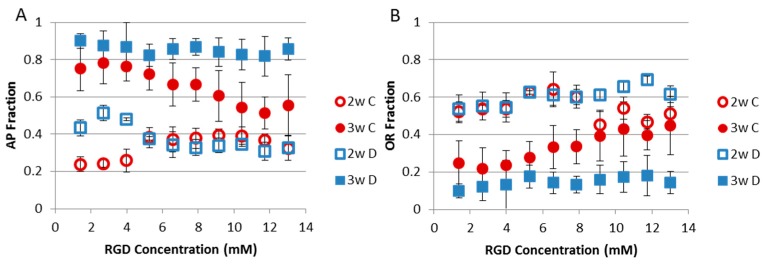
Effect of gradient culture condition on human mesenchymal stem cell lineage selection. (**A**) Fraction of cells expressing alkaline phosphatase, an osteogenic marker; (**B**) Fraction of cells with adipogenic vacuole staining in continuous culture, (**C**) which allows free access to cytokines secreted from cells across the gradient, and discrete culture, (**D**) which limits cytokine access to those secreted from cells in nearly similar RGD concentrations. Adapted and Reprinted with permission from [103]. Copyright 2013 American Chemical Society.

The effects of cellular crosstalk between positions is another critical design consideration in combinatorial method/high throughput systems. hMSC lineage choice was used to examine the effects of cellular crosstalk in a recent study [103]. Access to cell secreted cytokines was either freely allowed across an RGD concentration gradient hydrogel or restricted through sectioning and discrete culture of gradient sections in isolated tissue culture wells [103]. The study found that free access to cytokines from hMSC exposed to all test RGD concentrations favored adipogenic differentiation, while restricted access to only cytokines secreted from hMSC exposed to similar RGD concentrations favored osteogenic differentiation (Figure 4) [103]. This highlights to potential effect of cell secreted cytokines as a confounding factor in analysis of combinatorial method/high throughput systems, which can alter biological results. The effects of crosstalk can become even more complicated when more than one cell type is included in the combinatorial/ high throughput sample [40,48,117].

As technology advances and designs of combinatorial method/high throughput samples become more complex, isolating the effects of each test parameter on cellular response will be increasing important. As shown by the complex studies already in the literature, one component can dominate the cellular response masking the effects of the others if not properly managed [70]. Alterations in one test parameter can also modulate the response of another, providing apparently conflicting results in terms of biological response for the second parameter [88]. Expansion of temporal studies, pose additional complexity as the cellular differentiation state and even point in cell cycle can alter response. These technological advances will likely lead to utilization of more advanced biological outputs in combinatorial method/high throughput strategies. As the complexity of the biological outputs increases, the chances of identify more interconnected test parameters will also increase.

## 6. Concluding Remarks

Broader adoption of these combinatorial method/high throughput strategies is necessary is to efficiently optimize hydrogels and bring the promise of tissue engineering closer to fruition. The extent of how hydrogels influence cellular behavior has not yet been fully elucidated. Alterations in cellular response due to cellular differentiation states, species of cellular origin and culture type are just beginning to be understood [10,73,118,119,120]. Combinatorial method/high throughput strategies offer the ability to systematically develop the base of knowledge necessary for model development, which will finally enable the rational design of biomaterials to emulate key ECM factors governing cellular behavior. However, as the biology of cell-material interaction becomes better appreciated and the complexity of biological outputs advances with technology in combinatorial method/high throughput strategies, so must the level of material characterization. Post-fabrication, as well as during culture, material characterization must occur in order to keep pace with the biology in order to truly elucidate how the two systems (material and biological) alter each other over time. This will require the development of new material characterization procedures.

DOE, arrays and gradient samples, the three methods discussed in this review that have already been applied to hydrogel optimization, are just the first step in this movement toward systematic studies and rational design. Each has its own advantages and drawbacks when utilized in the optimization process, which makes it better suited for particular types of studies. However, they all allow for the optimization of multiple test parameters simultaneously using a systematic approach, which has been shown in each case to identify parameter interactions that traditional methods of hydrogel optimization have failed to identify. As the field advances, these strategies as well as new ones which have not been developed or adapted to hydrogel development will be utilized from discovery to final optimization in combination. They will become even more powerful tools for hydrogel design and optimization. Imagine the additional efficiency obtained from using DOE to dictate sample formulations in a miniaturized array. The results from those experiments could then be fine-tuned in gradient samples to obtain the final optimization. In fact, the cross utilization of multiple combinatorial method/high throughput strategies in a single study has already begun [108,121]. This should decrease everything from total development time to research cost. A move that will bring the hydrogel development process much more in line with the pharmaceutical industry and hopefully bring many more tissue engineering based treatments to the clinical application with greater speed.

## Abbreviations

The following abbreviations are used in this manuscript:

ECM	extracellular matrix
DOE	design of experiment
hMSC	human mesenchymal stem cells
HUVEC	human umbilical vein endothelial cell
hESC	human embryonic stem cell
